# Aspiration in Pneumothorax

**Published:** 1890-05-03

**Authors:** 


### SURGERY.

### ASPIRATION IN PNEUMOTHORAX.

It is usual to rely on the efforts of nature, aided by external pressure for the absorption of air in the cavity of the pleura, but it is only reasonable to suppose that the absorption of air, no less than of fluid, may be greatly accelerated by the removal by aspiration of the greater part of it. This procedure will not, however, we fear, be of any avail in phthisical cases in which the pulmonary pleura has been perforated by excavations, and is therefore not in a state permitting of healing, though in traumatic pneumothorax, including ruptures following violent respiratory efforts, as in asthma and whooping cough, in which the lung itself is healthy and capable of repair, it should certainly be performed, so soon as the wound in the lung may be assumed to have closed. M. Trosier, in a case of pneumothorax occurring during a violent attack of spasmodic asthma, thus aspirated $2\frac{1}{2}$ litres of air, and found that the remainder was absorbed in the course of the following day. M. Rendu did the same in a case of pneumothorax following whooping-cough, and Mr. Waller in one of unknown origin. In these the exciting cause had passed away, but M. Trosier's patient had, during the two years following the operation, several paroxysms of asthma no less severe, but without any return of the pneumothorax. In such cases an interval of rest from the attacks should be chosen for the operation.

